# Conceptualization of a Parasympathetic Endocrine System

**DOI:** 10.3389/fnins.2019.01008

**Published:** 2019-09-23

**Authors:** Jonathan Gorky, James Schwaber

**Affiliations:** Daniel Baugh Institute for Functional Genomics and Computational Biology, Thomas Jefferson University, Philadelphia, PA, United States

**Keywords:** parasympathetic, autonomic, endocrine, gut, vagus

## Abstract

We here propose a parasympathetic endocrine system (PES) comprised of circulating peptides released from secretory cells in the gut, significantly modulated by vagal projections from the dorsal motor nucleus of the vagus (DMV). While most of these gut peptides mediate well-described satiety and digestive effects that increase parasympathetic control of digestion (Lee et al., 1994; Gutzwiller et al., 1999; Klok et al., 2007), they also have actions that are far-reaching and increase parasympathetic signaling broadly throughout the body. The actions beyond satiety that peptides like somatostatin, cholecystokinin, glucagon-like peptide 1, and vasoactive intestinal peptide have been well-examined, but not in a systematic way. Consideration has been given to the idea that these and other gut-derived peptides are part of an endocrine system has been partially considered (Rehfeld, 2012; Drucker, 2016), but that it is coordinated through parasympathetic control and may act to increase the actions of parasympathetic projections has not been formalized before. Here only gut-derived hormones are included although there are potentially other parasympathetically mediated factors released from other sites like lung and liver (Drucker, 2016). The case for the existence of the PES with the DMV as its integrative controller will be made through examination of an anatomical substrate and evidence of physiological control mechanisms as well as direct examples of PES antagonism of sympathetic signaling in mammals, including humans. The implications for this conceptual understanding of a PES reframe diseases like metabolic syndrome and may help underscore the role of the autonomic nervous system in the associated symptoms.

## The DMV as the PES Controller

**FIGURE 1:**
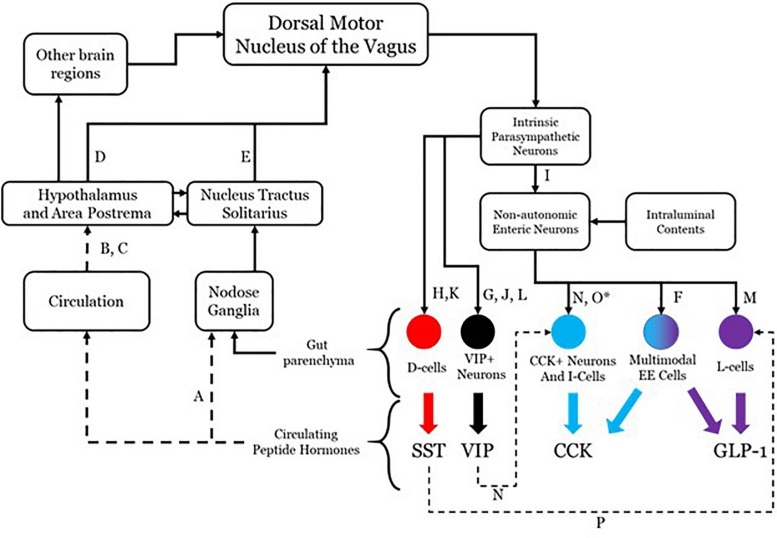
Anatomical connectivity of the proposed parasympathetic endocrine system. Each solid arrow represents direct synaptic neuronal connections and dashed lines represent humoral or indirect influence, but with documented influence. The afferent limb is shown on the left and the efferent limb shown on the right. The letters in the connectivity map identify examples of literature that supports the existence of the specific connection. A, Dockray (2013); B, Dockray (2012); C, Sandoval (2008); D, ter Horst (1984); E, Sawchenko (1983); F, Egerod (2012); G, Bitar et al. (1980); H, Greenberg (1993); I, Hayakawa (2006); J, Yuan et al. (2005); K, Ahrén et al. (1986); L, Kirchgessner (1989); M, Rocca and Brubaker (1999); N, Furness (2000); O, Liddle (2000) (^∗^permissive in human); P, Chisholm and Greenberg (2002).

Although there is evidence that more may participate, four effectors will be considered in this conceptualization: somatostatin (SST), cholecystokinin (CCK), glucagon-like peptide 1 (GLP-1), and vasoactive intestinal peptide (VIP). The system as hypothesized is shown in Figure 1 with annotations as to each aspect of the connectivity. While all four are used as neurotransmitters and do not readily cross the blood brain barrier (Banks and Kastin, 1985), their presence in the circulatory system is able to mediate brain function through receptors in the hypothalamus and area postrema (van der Kooy, 1984; Shaffer and Moody, 1986; Breder et al., 1992; Yamamoto et al., 2003; Arora and Anubhuti, 2006), both of which prominently project to the dorsal motor nucleus of the vagus (DMV) (Gray and Magnuson, 1987; Hyde et al., 1996; Zheng et al., 2005). This sets up a pathway by which DMV sensing for circulating peptide levels and their effects may be monitored and controlled. The DMV is capable of modulating secretion of each of these through direct and indirect means utilizing gut postganglionic and myenteric plexus neurons. The work on such vagal influence has not been fully investigated in any one species, so the literature cited here includes work done in rats, guinea pigs, dogs, and humans. VIP is secreted by a subset of secretomotor neurons under the influence of postganglionic parasympathetic neurons directly modulated by vagal efferents (Bitar et al., 1980; Kirchgessner and Gershon, 1989; Yuan et al., 2005). SST is released from D-cells and is also under the influence of postganglionic parasympathetic neurons with vagal efferent projections having a demonstrable influence (Ahrén et al., 1986; Greenberg, 1993; Chisholm and Greenberg, 2002). CCK and GLP-1 secretion occurs under direct modulation by enteric neurons, which themselves have their influence from the postganglionic parasympathetic neurons. There is evidence that GLP-1 secretion can occur as a direct consequence of vagal efferent activity (Rocca and Brubaker, 1999). Although no such linear pathway has been described for CCK release as a result of vagal efferent activity, there is molecular and anatomical evidence that an indirect pathway exists. This pathway contains enteric secretomotor neurons that produce CCK and interactions with enteric neurons and possibly postganglionic neurons.

The DMV efferent projections are capable of modulating the release of several peptides in the gut, including the four enumerated above. Each of these four peptides are released through a complex network of local (gut sensing) and central (vagal) mechanisms. It is possible that the release of all gut peptides, including but not limited to the four discussed here, results from generic vagal efferent activity [release of acetylcholine (Ach)]. There is even evidence that changing the firing frequency can modulate peptide release of DMV efferents, which itself may be influenced by the transcriptional milieu of ion channels (Guzman et al., 1979; Nishi et al., 1985). DMV efferent neurons have the potential to express several other transmitters and peptides, the effects of which are still incompletely understood, especially with regard to local effects on signaling of gut projections.

## Antagonism of the Sympathetic Nervous System

**FIGURE 2:**
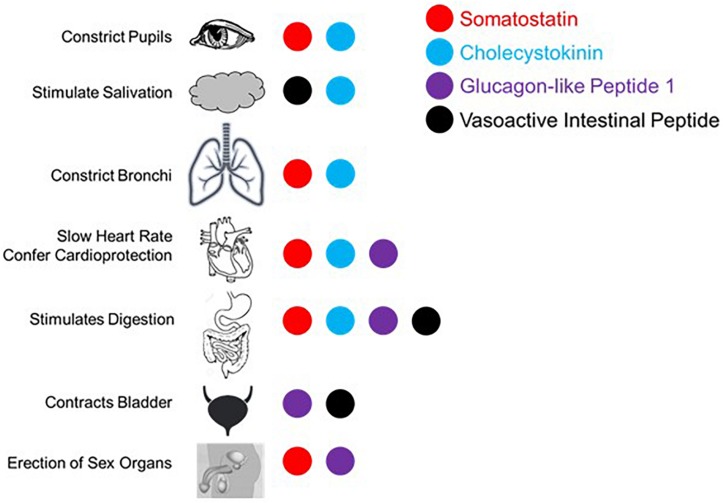
Summary of published effects of four selected gut peptides and their effects in augmenting parasympathetic function canonically effected via the vagus nerve and/or sacral parasympathetic projections.

The proposed parasympathetic endocrine system (PES) counterbalances the sympathetic nervous system broadly, not just with regard to digestion and orexigenic behavior. As the role of the four peptides examined here in digestion and satiety has been well-described (e.g., Arora and Anubhuti, 2006), we here focus on the other aspects of their control over visceral functions as they pertain to sympathetic antagonism. First, it is helpful to recall what the canonical effects of the sympathetic nervous system are. Sympathetic activation causes pupillary dilation, increased rate and contractility in the heart, bronchial dilation in the lungs, constriction of blood vessels generally, fluid retention and Na+ reuptake in kidney, urinary bladder relaxation, and activation of sweat glands, and is generally proinflammatory (Grebe et al., 2010; Kreibig, 2010). While not antagonistic to sympathetic activity, several PES peptides have the parasympathetic-like activity of enhancing erection induction, as will be discussed. What follows is evidence for sympathetic balancing in mammals from each of the PES effectors under consideration with these effects being summarized in Figure 2.

### Somatostatin

Somatostatin and neuronostatin, a closely related peptide derived from the same mRNA transcript, mediate broad sympathetic antagonism and parasympathetic potentiating effects. In the heart, SST reduces contractility in an Ach-dependent fashion (Franco-Cereceda et al., 1987; Yoshikawa et al., 1996) and directly antagonizes sympathetic adrenergic beta receptors (Murray et al., 2001). Neurostatin also diminishes contractility and has the net effect of reducing blood pressure when in circulation (Samson et al., 2008; Vainio et al., 2012). Central administration of SST causes apnea (Yamamoto et al., 1988) and local levels in the lungs result in bronchoconstriction (Barrios et al., 1987). SST also has broad anti-inflammatory effects (Hofland et al., 1999; Krantic, 2000; ten Bokum et al., 2000). In penile tissue, SST potentiates the effects of Ach induction of erection (Hedlund and Andersson, 1985). Even in the eye, SST alone causes minor pupillary constriction (Bito et al., 1982). Along with the effects mentioned above, SST is able to mimic the effects of parasympathetically mediated ischemic preconditioning in generating cardioprotection (Wang et al., 2005).

### Cholecystokinin

The cardiovascular effects of CCK (Lovick, 2009) include reduction of heart rate (Kaczyńska and Szereda-Przestaszewska, 2015). CCK is also a vasodilator, acting locally (Sánchez-Fernández et al., 2004) or directly antagonizing neurons in rostral ventrolateral medulla (RVLM) and caudal ventrolateral medulla (CVLM) that selectively mediate vasoconstriction (Sartor and Verberne, 2002, 2006). Systemically administered CCK causes dyspnea with large enough doses in adults (Bradwejn et al., 1998) and can even induce panic attack like breathing patterns (Shlik et al., 1997). In the kidney, CCK decreases Na+ reuptake and reduces vascular resistance (von Schrenck et al., 2000) along with decreasing renal inflammation (Miyamoto et al., 2012). It has more broad anti-inflammatory effects via enhancement of vagal signaling (Luyer et al., 2005). CCK causes pupillary constriction, but only in primates and humans (Bill et al., 1990).

### Glucagon-Like Peptide 1

In the lungs, GLP-1 stimulates macromolecule secretion, mimicking the effects of Ach, and increases pulmonary blood flow (Richter et al., 1993). In the kidney, GLP-1 inhibits Na+ reuptake, thus acting as a diuretic (Okerson and Chilton, 2012) and also induces diuresis in the bladder through contraction (Ahrén, 2004). Like SST, GLP-1 also enhances erectile function via direct binding of receptors in erectile tissue (Giagulli et al., 2015). GLP-1 has also been shown to mediate a robust cardioprotective response (Ban, 2010; Basalay et al., 2016).

### Vasoactive Intestinal Peptide

Like the other peptides, VIP is a potent vasodilator capable of decreasing heart rate and conduction velocity (Henning and Sawmiller, 2001). Interestingly, there are more VIP receptors in the right ventricle compared with the left, although the functional implications of this is not well understood (Henning and Sawmiller, 2001). In the kidney, VIP increases Na+ excretion (Rosa et al., 1985) and can induce erection or vaginal lubrication (Sjöstrand et al., 1981; Ottesen et al., 1984, 1987; Hedlund and Andersson, 1985). There are several ways in which VIP mediates anti-inflammatory effects (Pozo et al., 2000; Ganea and Delgado, 2002; Delgado et al., 2004): inhibits mast cell degranulation (Tunçel et al., 2000), decreases lymphocyte proliferation in Peyer’s patches (Stanisz et al., 1986), induces Treg and regulatory dendritic cell expansion (Chorny et al., 2005), and is generally immunosuppressive in aqueous humor (Taylor et al., 1994). In contrast to the other peptides considered here, VIP is sympathomimetic in the lungs, antagonizing bronchoconstriction (Barnes and Dixon, 1984). On the balance, VIP has parasympathetic-like activity in spite of the respiratory function described here.

## Reconsidering Metabolic Syndrome

There are likely more effectors of the PES than the four peptides explored above with partial or complete sympathetic antagonism. What makes this idea more than a collection of peptide functions is that it is coordinated by DMV projections. Reconsideration of disease processes in this context as a system may provide a foundation for new treatment approaches. Autonomic dysfunction accompanies many metabolic syndromes, including obesity and type II diabetes. There therefore may be some aspects of metabolic syndrome that are mediated, or at least modulated by autonomic functions via this parasympathetic endocrine system.

**FIGURE 3:**
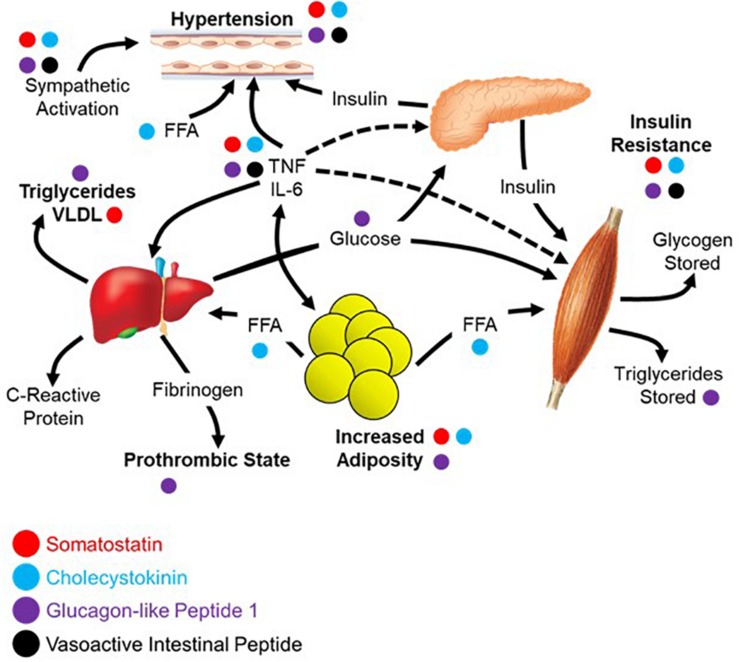
Parasympathetic endocrine effects on multi-organ network interactions that give rise to metabolic syndrome. Aspects of metabolic syndrome that are inhibited by the four selected gut peptides as was summarized in the text of the section “Reconsidering Metabolic Syndrome.” The relationships between different aspects of metabolic syndrome are recreated from the work of Eckel et al. (2005).

The diagnostic criteria for metabolic syndrome includes a combination of at least three of the following: abdominal obesity, hypertension, hyperglycemia, increased triglycerides, and decreased high-density lipoprotein (HDL) cholesterol (Alberti et al., 2005; Opie, 2007). The four peptides highlighted in this review each contribute to negating the effects of metabolic syndrome across these five criteria as shown in Figure 3, the underlying network being based largely on the work of Eckel et al. (2005). Abdominal obesity can be reduced/prevented through circulating SST (Lustig et al., 1999; Boehm and Lustig, 2002; Boehm, 2003), CCK (Pi-Sunyer et al., 1982; Matson and Ritter, 1999; Neary and Batterham, 2009), or GLP-1 (Näslund et al., 1999; Day et al., 2009; Astrup et al., 2012). Hypertension can be ameliorated by all four peptides focused upon here; SST (Rosenthal et al., 1977; Carretta et al., 1989), CCK (Kagebayashi et al., 2012), GLP-1 (Wang et al., 2013; Katout et al., 2014), and VIP (Gao et al., 1994). All four peptides contribute to lowering fasting plasma glucose through a variety of mechanisms including stimulation of insulin secretion and inhibition of glucagon secretion; SST (Gerich et al., 1974, 1975; Wahren, 1976; Vergès, 2017), CCK (Cheung et al., 2009; Irwin et al., 2015), GLP-1 (Nauck et al., 1993; Toft-Nielsen et al., 1999; Irwin et al., 2015), and VIP (Kato et al., 1994). Effects on triglycerides and HDL cholesterol have less evidence to date, yet it has been demonstrated that GLP-1 can lower triglycerides (Qin et al., 2005; Meier et al., 2006) with CCK recently shown to have similar effects on absorption in a preclinical model (Plaza et al., 2018). Of the four, only GLP-1 can increase HDL cholesterol, although it appears to be mediated through a variety of other mechanisms as opposed to a direct effect on the production and processing of cholesterol (Ponzani et al., 2016).

Apart from the direct symptoms of metabolic syndrome, there is a broad sympathetic dominance across multiple organ systems (Tentolouris et al., 2006, 2008). This includes cardiovascular problems like resting tachycardia, reduced heart rate variability, and decreased baroreflex sensitivity (Garruti et al., 2012; Verrotti et al., 2014). Metabolic syndrome causes erectile dysfunction with a high incidence of comorbidity (Gündüz et al., 2004; Bal et al., 2007). Also in metabolic syndrome, there is a markedly decreased release of multiple gut peptides (Verrotti et al., 2014). It is possible that the diminished gut peptide release contributes to the dysautonomia and sympathetic dominance through absence of antagonism. This may be due to physical changes in the gut or perturbations in the sensory mechanisms that otherwise mediate their release. If this is the case, it might be possible to replace these peptides and treat the autonomic symptoms. There is an example of this in a clinical trial showing that the use of SST analog treats diarrhea and orthostatic hypotension in patients with diabetes (Dudl et al., 1987). Moving forward, it may be helpful to assay for circulating peptide levels and use them either a biomarkers of disease or as indications to initiate peptide replacement therapy. It is likely that replacing the milieu of peptides rather than one or the other will be required for maximal clinical benefit.

## Conclusion

The requirements of the PES laid out here were that it has a central controller, broadly counterbalances sympathetic effects, and can help explain disease pathology. The evidence provided suggests that there is a parasympathetic endocrine system that is coordinated by the DMV. The power lent by this concept derives from the network coordination of the four peptides discussed here and many additional circulating gut factors at the level of the DMV where there can be integration of peripheral and central neuronal inputs and orchestration of multiple gut endocrine activities. This also enables identification via disease markers and interventions aimed at treating a network of factors at the central or peripheral level. The conceptualization of physiology laid out here goes beyond the boundaries of traditional medical specialties like gastroenterology, neurology, or cardiology and instead requires a systems approach to health and medicine. As the ability to deal with human health in a more comprehensive and complete way matures in the era of big data, clinicians must be ready to incorporate the growing complexity of the body as a system in the design and implementation of therapeutic intervention.

## Author Contributions

JG developed the main concepts and was the primary writer of the manuscript. JS provided the significant editorial contributions.

## Conflict of Interest Statement

The authors declare that the research was conducted in the absence of any commercial or financial relationships that could be construed as a potential conflict of interest.
